# Supplementary material for: The causal role of circulating inflammatory markers in osteoporosis: a bidirectional Mendelian randomized study
Source: Front Immunol. 2024 Jul 18;15:1412298. doi: 10.3389/fimmu.2024.1412298 (PMC11291241; doi:10.3389/fimmu.2024.1412298)
Supplement: Supplementary file 1 [file DataSheet_1.zip › supplementary material/CIM-OSTEOPOROSIS /GCST90274832.csv_leave_one_out.pdf]

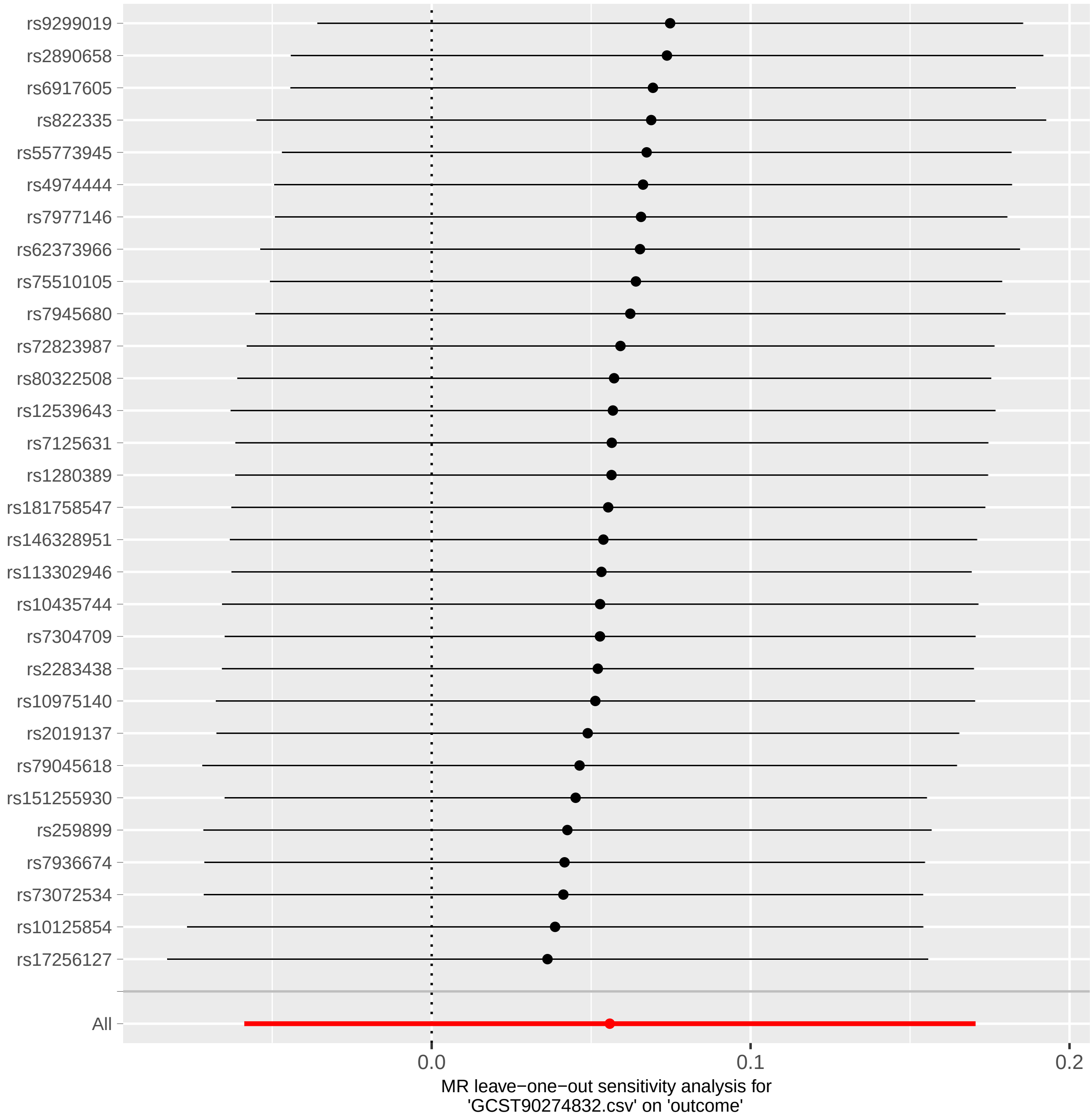

rs9299019
rs2890658
rs6917605
rs822335
rs55773945
rs4974444
rs7977146
rs62373966
rs75510105
rs7945680
rs72823987
rs80322508
rs12539643
rs7125631
rs1280389
rs181758547
rs146328951
rs113302946
rs10435744
rs7304709
rs2283438
rs10975140
rs2019137
rs79045618
rs151255930
rs259899
rs7936674
rs73072534
rs10125854
rs17256127
All
0.0
0.1
0.2
MR leave−one−out sensitivity analysis for
'GCST90274832.csv' on 'outcome'
